# Annotations

**Published:** 1905-11-18

**Authors:** 


### ANNOTATIONS.

### The Church and the Medical Profession.

The fact that on Monday evening a member of the Christian Science Board of Lectureship, of Boston, U.S.A., commenced a series of lectures in the Queen's Hall, intended to place the subject of Christian Science before the public "in an authoritative form," emphasises the importance of the utterances of the Bishop of London on the previous Saturday. The claims which Mr. Young, from America, urges on behalf of faith healing as a substitute for medical treatment were demolished in advance by Dr. Winnington Ingram, whose condemnation was all the more striking because he recognises in Christian Science an element of truth. He admits that by means of the faith, the hope, and the courage which belief in the verities of Christianity can inspire, the bodily condition of a sufferer from disease may, in many cases, be greatly improved. But while, in that sense, he is in agreement with the Christian Scientist, he affirms that "those who go on to other points in Christian Science are degrading the real truth with a gigantic heresy," and he proceeded to warn any persons who think that they have special gifts never to try to exercise them "apart from the medical profession." The American lecturer, who glorifies Mrs. Eddy, puts forward faith healing as an alternative to seeking the advice of the medical man. The Bishop of London insists that the medical man is a necessity in the case of illness, and he declares that the Church considers the healing act of the medical profession as a sacred thing. Moreover, he says "that when he was ill he felt that the doctor who came to him was as much sent by Jesus Christ as the clergyman who called to visit him." This is an attitude which will be warmly appreciated by all who regard the practice of medicine from the highest and noblest point of view. So long as the Church welcomes the members of the medical profession as co-workers with the clergy in work which was consecrated for ever by the teaching and the example of the Founder of Christianity, we are sure that the appeal of the Bishop to the former to freely acknowledge the right of the latter to a place in the sick-room will be greatly conceded. There is no desire on the part of doctors to exclude the clergy from the sick-rooms of either the wealthy or the poor, so long as they exercise the tact and discretion which the Bishop himself describes as indispensable.

### Feeble-Minded Mothers.

Periodically there arises among those interested in poor-law questions the matter of illegitimate children and their mothers. The growing humanity of our age has vastly improved the maternity wards of our large workhouses, and now the question arises: Are we, by making such provision for mothers and children as no woman in the class to which workhouse inmates belong, or in classes very much above that, can hope to enjoy in her hour of trial, putting a premium on immorality? It is difficult to avoid an affirmative answer. Yet no one would wish to solve the difficulty by taking any step which would render motherhood more dangerous. And it must be remembered that, for various reasons, there is an exceptionally large proportion of difficult confinements among the cases delivered in the maternity wards of workhouses. It is not by means of neglect or even lack of comfort that we must try to limit the evil. Some boards of guardians ask for powers of detention of the mothers of illegitimate children for periods varying from a year to a lifetime. This is to make unlegalised motherhood a crime, and it is doubtful if the law would consent to such a step. In one way, however, an improvement might be effected. If it was allowed to guardians to detain feeble-minded women a great deal of mischief, both to individuals and the race, might be saved. Of the women who return again and again to the maternity wards of the workhouse a considerable proportion are morally irresponsible, and to keep these under control would be a kindness both to themselves and to the unfortunate children who now inherit their weaknesses.

### Mistaken Diagnosis in Infectious Illnesses.

At a meeting of the Metropolitan Asylums Board held on Saturday the subject of mistaken diagnosis was aired once more. A little while ago the Board received a letter from the Holborn Borough Council calling attention to an alleged expenditure of about £60,000, which was said to have been occasioned by the admission into the Board's hospitals, during the last five years, of about 10,000 cases not suffering from diseases for which those hospitals were provided. This accusation (for the letter appears to have been intended as an accusation), in the meantime, has been under the consideration of the Hospitals Committee, who now report that the extra cost to which the Board has been put by the admission of those patients does not exceed an average sum of £3,200 per annum, as contrasted with the £12,000 estimated by the Borough Council of Holborn. The Committee further add that the delay which would be entailed by ensuring certainty of diagnosis in all cases before sending the patients to the isolation hospitals would lead inevitably to spread of infection, and so bring about that very thing which it is the object of the Board to prevent. This is a perfectly reasonable explanation. In scarlet fever, for example, it would be absurd to keep a suspected case under conditions where isolation was impossible until positive signs of the complaint appeared. As every medical man knows well, cases of scarlet fever frequently occur in which the patient presents no conclusive proof of the nature of his illness. In such an instance the doctor has to rely and act solely upon probabilities in order to do his duty to the public and to the patient. The wonder is that, under such circumstances, mistakes are not far more numerous than the records of the fever hospitals indicate. Disease is a process, and it is about as easy to make an early and correct diagnosis in every instance as it would be to foretell the result of a horse race merely from an instantaneous photograph of the competitors just after the start.

